# Features that hindered the capacity development of a national prostate cancer service

**DOI:** 10.3389/frhs.2023.1173143

**Published:** 2023-07-18

**Authors:** Ashleigh Lauren Ward, Susanne Cruickshank, Liz Forbat

**Affiliations:** ^1^ Directorate of Nursing, NHS Forth Valley, Larbert, United Kingdom; ^2^ School of Medicine, Veterinary and Life Sciences, University of Glasgow, Glasgow, United Kingdom; ^3^ The Royal Marsden NHS Foundation Trust, London, United Kingdom; ^4^ Faculty of Social Sciences, University of Stirling, Stirling, United Kingdom

**Keywords:** qualitative research and analysis, survey research and questionnaire design, clinical practice patterns/guidelines/resource used/evidence based practice, comparative health systems/international health, health care organisations and systems, nursing, medical decision-making, cancer service improvement

## Abstract

**Introduction:**

In Scotland, prostate cancer services have struggled to meet demand, and urological cancer services have missed Scottish Government waiting time targets to a greater extent than other cancer services. This study provides understanding of the capacity development needs of a national prostate cancer service including why the service had been unable to adapt to meet demand and how capacity could be developed.

**Methods:**

Delphi technique was applied to a purposive sample of prostate cancer clinicians working across Scotland between 2015 and 2017. Interviews were conducted with healthcare professionals involved in delivery of care to people with prostate cancer including General Practitioners, followed by questionnaires which were distributed to Specialist Nurses, Oncologists and Urologists involved in delivering specialist prostate cancer services within NHS Scotland. Findings are reported from interviews analysed using a directed approach to content analysis, followed by three rounds of iterative online questionnaires analysed using descriptive statistics.

**Results:**

Reform is needed to meet demand within prostate cancer services in Scotland. Barriers to capacity development included: lack of shared understanding of quality of care between policy makers and healthcare professionals; lack of leadership of service developments nationally and regionally; and difficulties in drawing on other capacities to support the service. Cohesive working and a need for efficient training for nurse specialists were needed to develop capacity. Consensus was reached for development of national working groups to set standards for quality care (100% agreement) and further development of existing regional working groups (100% agreement) to implement this care (91% agreement), which should include input from primary and community care practitioners (100% agreement) to meet demand.

**Discussion:**

This work provides important understanding of barriers and facilitators to service development across a national service, including highlighting the importance of a shared vision for quality care between policy makers and healthcare professionals. Mechanisms to support service change are identified.

## 1. Introduction

Health systems report varying levels of success in developing health services to manage demand following the emergence of COVID-19 (1–4). Recovery and remobilisation plans following the pandemic have led to further significant investment in health service development (5, 6), offering an opportunity to improve health systems towards a “new normal” for healthcare delivery rather than reverting systems back to their pre-pandemic forms (7, 8). This paper presents findings from a study that identified barriers and facilitators to development of a national cancer service to support successful development of health services.

In Scotland, incidence of prostate cancer has increased steadily over a number of years (9) and Scottish prostate cancer services have been unable to adapt to meet demand. Most evidently, the quality of cancer care was measured nationally using waiting time targets in Scotland (10, 11), and urological services have missed these waiting time targets to a greater extent than any other cancer care service (12). Further, capacity shortage within Scottish prostate cancer services resulted in delayed adoption of pre-biopsy MRI following updating of NICE guidelines in 2019. Prostate cancer services in Scotland have been identified as lacking capacity to deliver quality prostate cancer care in line with NHS Scotland cancer policy (11, 13). With no literature explaining why prostate cancer services in Scotland had been unable to develop to meet demand, this study was guided by limited availability of literature and existing datasets.

This study gained understanding of the capacity development needs of the NHS Scotland prostate cancer service. Specifically:

1. How is use of the NHS Scotland prostate cancer service predicted to change?
2. Why has the NHS Scotland prostate cancer service been unable to meet demand?
3. How should capacity be developed within the NHS Scotland prostate cancer service to meet demand?

## 2. Methods

### 2.1. Study design

**Figure 1:**
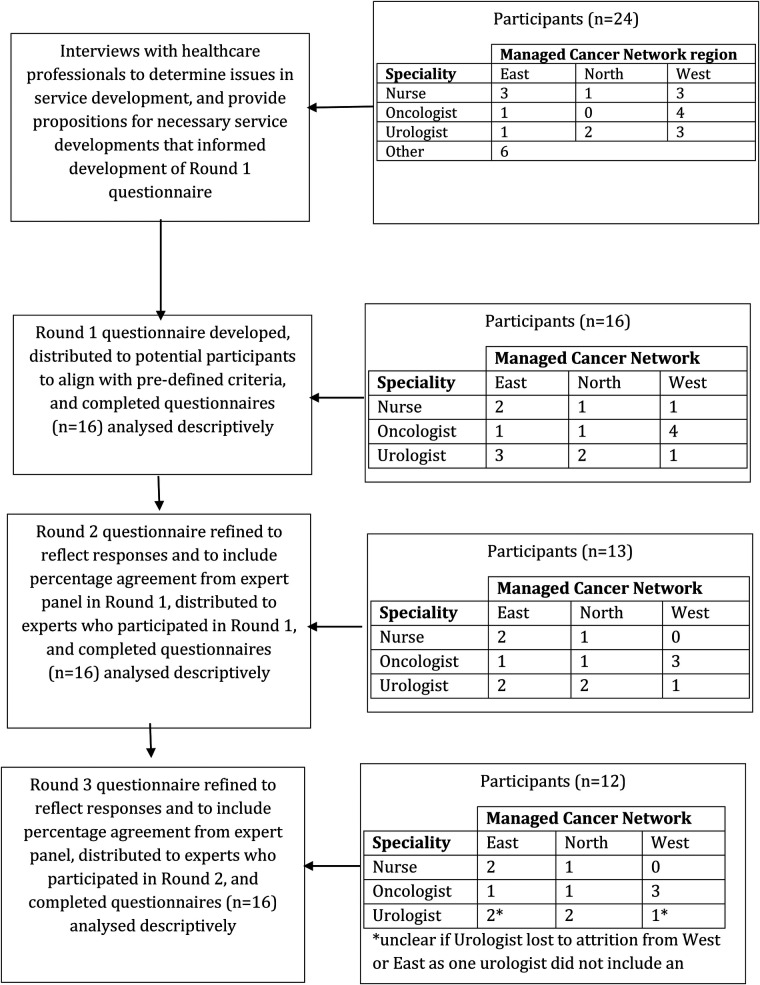
Flowchart of Delphi consensus steps.

Delphi technique was used to gain understanding of the capacity development needs within the Scottish prostate cancer service from a pragmatic, pluralist perspective. Delphi technique is a commonly used methodology in healthcare research (14) and provided a feasible framework for exploiting existing experience of prostate cancer services in Scotland to facilitate consensus on capacity need. When using Delphi, consensus is generally facilitated across two key steps. Firstly, a range of methods are used to generate ideas or determine issues related to the research problem. Then, findings are used to inform development of closed-question questionnaires that are distributed, analysed, revised and re-distributed iteratively until consensus is reached, attrition occurs, or over a pre-determined number of questionnaire “Rounds”. In this study, interviews with healthcare professionals were followed by an iterative questionnaire process with urologists, oncologists and nurses across a multiphase study. A flowchart of this study design is provided in Figure 1.

### 2.2. Setting

Within Scotland, the Scottish Government Directorate for Health and Social Care implement healthcare policy to direct healthcare development and are responsible for the administration of the NHS (15). Regional responsibility of healthcare is then devolved to 14 Health Boards who deliver care in collaboration with 32 local authority areas across Scotland. The delivery of disease-specific, specialised care is coordinated by three Managed Cancer Networks (MCN) in the East, North and West of Scotland, who are directly answerable to Health Boards (16). Each Health Board must meet local need (17) and so, service design and priorities differ across regions (18). As a result, some roles or services exist in one Health Board that do not exist in another, and there is a general lack of clarity on how each service was composed including the disciplines and roles present.

### 2.3. Interview participant sampling and recruitment

All healthcare professionals with an interest in prostate cancer serviceswithin NHS Scotland were invited to participate across all Managed Clinical Network (MCN) regions to ensure that the experience captured was reflective of the needs of the service across Scotland. Participants were invited to interview via email using an extensive list of healthcare professional contact details which had been collated from the research funder, professional networks, national conferences and networking events. Snowball sampling was also used. Recruitment continued until a diverse population has been interviewed and no new information was apparent (19).

### 2.4. Interview data collection

A structured interview schedule was developed to elicit a comprehensive understanding of service need. Scottish prostate cancer service use trends (20) were summarised to interviewees who were then asked four questions: (i) how do you explain this change in usage, (ii) how do you expect this to change in the future, (iii) what impact will this have on service delivery and organization, and (iv) how can services better plan to accommodate this change? Then, to ensure all service developments were accounted for, participants were asked to predict the main service development issues in the coming 10 years, and the impact of developments that were currently being implemented, such as the implementation of robotic prostatectomy technologies. Where participants had limited understanding of all parts of the service, participants were asked to describe their role within the Service and were asked only questions from the interview schedule relevant to their role.

### 2.5. Content analysis

A directed approach to content analysis was used to enable the understanding, construction, and development of the participant's perspective only (Bengtsson, 2016). Analysis was structured around a framework (21) developed from the known structure and usage of the service (Ward, 2020). Content analysis has proven useful in guiding analysis and reporting of findings from similar types of exploratory research (22), and is an “unobtrusive” method of data analysis (23).

All interviews were transcribed verbatim excluding non-speech and intonation sounds such as laughter, emphasis and pauses. Data were coded to the framework using NVivo (24), which allowed the coding process to stay rooted in the service and minimised opportunity for meaning or context being inferred from other sources. The framework was then developed to fit all relevant data. Relevant data were then condensed to *meaning units* by describing each datum in fewer words, but without losing its meaning (25). Mind mapping tool, GoConqur, was then used to map meaning units around codes. Mind mapping has been shown to be reflective of thought processes in qualitative analysis, which are usually non-linear and random (26) so data visualisation helped with categorisation. Further, as the first step in development of themes involves grouping similar meaning units (27), the data visualisation resulting from the initial mapping exercise proved useful in identifying similarities in meaning units between codes. The meaning units that emerged from the data around each service or code, provided understanding of the capacity development issues of that service, which were then compared to support understanding of the service.

### 2.6. Questionnaire participant sampling and recruitment

Healthcare professionals were recruited across all MCN regions. Given the focus of questionnaires on service design, recruitment during this phase was limited to specialist nurses, oncologists, and urologists only, to ensure that participants had the relevant expertise needed to complete questionnaires. Participants were invited to interview via email and were encouraged to forward the invite to participate to relevant colleagues.

### 2.7. Questionnaire development

Analysis of interview data was used to construct the initial questionnaire. An *a priori* decision was made to collect data using three iterative questionnaire rounds to prevent participant fatigue (28). An online survey platform was used to distribute the questionniare.

The Round 1 (R1) questionnaire was structured around three topics: the organisation of secondary and tertiary care, multidisciplinary working, and patient follow-up to include all service developments proposed by healthcare professionals at interview for diagnosis or treatment services; propositions given in the interview data were reduced to its simplest form before inclusion, for example, “*diagnostic services should be a one stop shop*” and “*orchidectomy should be discussed as a treatment option with patients*”.

Following R1, Round 2 (R2) and Round 3 (R3) questionnaires were restructured to reflect service design and the emerging consensus, and group feedback was provided to individuals within the questionnaires. R2 topics were the multidisciplinary team, diagnostics and active surveillance, radical treatment, and palliative oncology and included all propositions that had not reached consensus and any new propositions proposed by participants in R1. Participants were asked in comment boxes why they agreed or disagreed with each proposition to gain understanding of potential barriers to service development.

R3 topics were service delivery, the diagnostic pathway, active treatments, and palliative treatments. As little guidance is provided on the purpose of, or how to develop an R3 questionnaire (29, 30), the R3 questionnaire was used to ensure that (i) all topics of consensus were compatible, and (ii) to re-evaluate topics of dissensus considering the emerging consensus. To do this, statements that had reached consensus in either R1 or R2 were grouped and presented as a single proposition. For example, the following statement grouped 9 propositions that had reached consensus in R1 or R2, and asked participants whether they agreed or disagreed:

“*The management of side effects requires a dedicated team that is nurse- led. This team should be responsible for the treatment and management of erectile dysfunction, incontinence, bladder irritability and bowel issues across both surgical and radiotherapy services as required. This team should also be involved with patients both pre and post treatment and should be organised around tertiary and sub-specialist services*.”

In addition to shortening the length of the questionnaire, this afforded healthcare professionals the opportunity to consider the emerging consensus as a service and not as a series of isolated propositions. Relevant statements that had not reached consensus in R1 or R2 were then listed to allow participants to consider these alongside the proposed service.

During development of the R3 questionnaire it became apparent that no mechanism was being identified that could support the service to develop. Interview data, questionnaire comments and the emerging consensus were reviewed and a section was added to the end of the R3 questionnaire titled “*further consideration*” to propose mechanisms of capacity development within the service.

A variety of question styles were used to suit each question asked: participants were provided with multiple propositions and asked to select the proposition that they most agreed with; participants were asked to select all relevant propositions; five-point Likert scales (R1 only); and open-ended questions were used in the final section of the questionnaire. Each question also included the option to select “*not relevant to my expertise*” and comment boxes were included after each question to collect open feedback, disagree with questions asked and suggest new propositions.

### 2.8. Piloting questionnaires

To ensure clarity in questions asked without introducing bias or impacting on recruitment to the study, the R1 questionnaire was piloted, which resulted in two changes in addition to minor rephrasing of statements.

### 2.9. Questionnaire data analysis

Consensus was quantified as ≥80% agreement and dissensus was quantified as <80% agreement. Using these thresholds, data were categorised as “statement accepted” (80%–100% agreement), “dissensus” (21%–79% agreement), and “statement rejected” (0%–20% agreement).

## 3. Results

### 3.1. Participants

As the service was composed of a diverse range of healthcare professionals, gaining an understanding of the full complement of healthcare professionals working within the service was challenging. Participants at interview identified very small numbers of healthcare professionals specialising in prostate cancer care outside of nursing and medicine, and so care was taken in reporting of results to ensure participant anonymity.

24 healthcare professionals were interviewed from across Scotland (Figure 1). 21 were interviewed in person, two via telephone and one participant provided written responses.

In questionnaire rounds, 16 healthcare professionals participated from across Scotland (Figure 1). Of these participants, 10 had participated in the interview phase of this study.

### 3.2. Findings from interviews

#### 3.2.1. Service reform

Healthcare professionals identified substantial capacity shortage across the entire service and raised concerns that finite resources had not increased at the same rate as incidence or treatment usage. Participants had “*redesigned as much as [they] probably [could], and we’re struggling”* (Participant 1) meaning that “*radical changes that are needed to say, new models of working… making small changes is important… but at a fundamental level for us to deal with capacity we need to look at bigger changes*” (Participant 9). Any predicted decline in treatment usage was predicted to be the result of changing patterns of presentation, the implementation of technological advances, or the integration of new technologies, and not due to a decrease in the number of patients requiring treatment. However, these changes were unlikely to be imminent, and so all healthcare professionals were anticipating increased demand.

#### 3.2.2. Shared vision

Though healthcare professionals and government were striving for a quality service, a shared vision for quality care was lacking. For example, healthcare professionals described government measures of quality such as waiting time targets as “*stifling*” (Participant 22) service development, were understood to be without scientific rationale, diverted focus from the development of quality services, and “*compromise[d] innovation and change in practice*” (Participant 17). With limited evidence to inform service development, healthcare professional's understanding of quality care was found to be a primary driver in service development, and services were developed only where healthcare professionals believed that the quality of the service could be maintained or improved rather than to meet government objectives as “*you don’t want to arrange [treatment] too early in the pathway to feed, to, to meet a necessity rather than what is correct*” (Participant 1). Healthcare professionals believed that their approach to quality care was now being “*validated*” (Participant 16) by recent evidence.

#### 3.2.3. Leadership

At interview, a lack of leadership or oversight of service developments resulted in wasted capacity as “*everybod[y] reinvent[ed] that wheel*” (Participant 5). Service developments were generally designed and implemented within each Health Board, putting significant pressure on local services to resolve capacity shortage issues without the support of the wider service to know “*what works, what doesn’t work*” (Participant 5). In part, this local approach was perpetuated within the structure of healthcare within Scotland, for example:

*“Why do we have 14 different area drug and therapeutic committees that decide what should and should not be prescribed in a way that leads to post, post book prescribing. In Scotland 5 and a half million people should be one overarching strategic approach to healthcare that is broadly standardised across Scotland.”* Participant 16

Healthcare professionals were unified in wanting more oversight of service developments, and a unified approach to service development. MCNs were identified as best placed to “*shape and design*” (Participant 16) service developments, but were described as underdeveloped, often guided by the larger Health Boards within the region and with little power to make a difference as they were “*just a titch of a role that has no teeth… it's just token shop I think”* (Participant 17). Further, each MCN was in a different stage of development, with the most advanced, considered “*integrated*” (Participant 4) and the least advanced considered “*broken*” (Participant 4), further complicating possibility of oversight of service development.

#### 3.2.4. Cohesion

When service development issues were described, specialities were described as working in isolation and often competing for resource, resulting in “*petty politics*” (Participant 22) and hindered the ability of service to plan ahead and develop, as specialisms put the needs of their part of the service before the needs of the service as a whole:

“*If we could get people talking together, staff, on the way forward, it would be great. And it has been tried, but it never works, sadly because they all want the best for their patients, whether that's surgery or oncology, so they’re always fighting. Sad. Sad but true.”* Participant 14

And healthcare professionals, including some within the speciality, raised concerns over one speciality being dominant within the service and the subsequent risk of “*bias*” (Participant 24) in patient care. Many healthcare professionals identified that “*more together working*” (Participant 5), and more “*cohesive working to plan ahead*” (Participants 14) was needed.

#### 3.2.5. Recruitment and training

More nurse specialists and further development of the nurse specialist and allied healthcare professional roles was found to be integral to capacity development within the service. However, recruitment and training were significant concerns for service development as “*how do you broadly train someone for a specialist role?”* (Participant 12). Currently, training of nurse specialists required significant investment of service capacities over years. Further, concerns were raised because of changes in undergraduate curricula in some disciplines and healthcare professionals identified specialist vacancies elsewhere in the UK that recruiters had been unable to fill.

#### 3.2.6. Lack of other capacities to use

Healthcare professionals were being encouraged by Scottish Government to use capacities of other services and were also looking to identify available capacities beyond NHS services that could be used. Policy drivers encouraged use of primary care services to alleviate capacity within the service. However, healthcare professionals raised concerns over General Practitioners (GP) capabilities to fulfil their current roles, namely PSA screening and general patient support during and following specialist treatment, and it was unlikely that this would change. Reasons given for this included short appointment times, “*their current numbers and as well as that, to educate them. A GP might have 2 patients with prostate cancer in his books or whatever, you can’t expect them to be an expert on that*” (Participant 5), pressures to adopt roles from other specialities, “*non-clinical issues really, the paperwork, chasing the points and all this sort of stuff”* (Participant 8). Healthcare professionals interviewed were divided over whether follow-up with primary care specialists was appropriate as “*it's just as expensive to have a GP seeing a patient as it is to have [a medical consultant] seeing a patient, where it's, if you can have in the hospital, you’ll have speciality doctors, you can have nurse practitioners where that is their clinical expertise”* (Participant 4); whilst some healthcare professionals believed that follow-up in community supported “*normal life*” (Participant 5), other healthcare professionals believed that “*they’ve had specialist treatment and they should have specialist follow-up… its quite a specialist follow-up regime*” (Participant 10). Further, healthcare professionals recognised that patients had capacities that could be used to meet demand and were “*expecting more from our patients*” (Participant 14). However, healthcare professionals were also concerned that not all patients would manage greater responsibility in their care and were hesitant to adopt individualised follow-up protocols.

### 3.3. Findings from the consensus questionnaires

Data are presented in Tables 1–4. In each table, statements accepted are presented first, followed by statements where dissensus remained and finally, statements that were rejected. Percentage agreement with the statement is also provided for each Round. The number of participants who responded to each statement excluding the number of participants who selected “not relevant to my experience” is given as “n”.

**Table 1:** Consensus on leadership and cohesion of service development.

Statement	Round 1 (*n* = 16)	Round 2 (*n* = 13)	Round 3 (*n* = 12)
Statements accepted			
1. MCNs should be further developed	93% (*n* = 14)	—	—
2. Within prostate cancer services, Managed Cancer Networks need to be responsible for…			
a. …auditing services	100% (*n* = 16)	—	100% (*n* = 12)
b. …planning ahead for the introduction of advances in treatment	94% (*n* = 16)	—	100% (*n* = 12)
c. …keep government up to date with advances in treatment	94% (*n* = 15)	—	100% (*n* = 12)
d. …managing the impact of centralisation on Health Boards	93% (*n* = 14)	—	100% (*n* = 12)
e. …the delivery of equitable care across the region	87% (*n* = 16)	—	100% (*n* = 12)
f. …have a role in funding and governance	71% (*n* = 14)	80% (*n* = 10)	100% (*n* = 12)
3. Methods of integration and communication between urology and uro-oncology services should be improved	—	83% (*n* = 12)	100% (*n* = 12)
4. [Some] tasks should be the responsibility of the relevant speciality and not be the responsibility of the MCN	—	89% (*n* = 9)	—
5. Prostate cancer services require the development of Scotland-wide surgery, radiotherapy and hormone therapy/chemotherapy working groups	—	—	100% (*n* = 12)
6. These groups should be responsible for the design of speciality specific “gold standard” care for patients living in Scotland	—	—	100% (*n* = 12)
7. These working groups should be responsible for communicating with Managed Cancer Networks	—	—	92% (*n* = 12)
8. Managed Cancer Networks should be responsible for the regional multidisciplinary implementation of ‘gold standard’ care	—	—	91% (*n* = 12)
Dissensus			
9. Prostate cancer services should be led by MCNs	63% (*n* = 16)	—	—
10. Prostate cancer services should be led Scotland-wide	31% (*n* = 16)	—	—
11. Urology and uro-oncology services should…			
a. …work separately, but with better lines of communication	44% (*n* = 16)	—	—
b. …work in the same capacity that they currently do	31% (*n* = 16)	—	—
c. …work together within the one overarching service	25% (*n* = 16)	—	—
Statements rejected			
12. Prostate cancer services should be led by Health Boards	6% (*n* = 16)	—	—

**Table 2:** Consensus on recruitment and training of nurse specialists.

Statement	Round 1 (*n* = 16)	Round 2 (*n* = 13)	Round 3 (*n* = 12)
Statements accepted			
1. Active surveillance should incorporate counselling services	91% (*n* = 11)	-	100% (*n* = 12)
2. Support services should be involved with men prior to receiving treatment	90% (*n* = 10)	-	92% (*n* = 12)
3. Follow-up clinics should include a supportive role	100% (*n* = 13)	-	91% (*n* = 11)
4. Clinics are currently not flexible enough to provide a supportive role to patients	82% (*n* = 11)	-	91% (*n* = 11)
5. A service is required to manage erectile dysfunction	87% (*n* = 16)	-	92% (*n* = 12)
6. A service is required to manage incontinence	94% (*n* = 16)	-	92% (*n* = 12)
7. Erectile dysfunction and incontinence support to be made available to radiotherapy patients	94% (*n* = 16)	-	92% (*n* = 12)
8. A service is required to manage bladder irritability	69% (*n* = 13)	91% (*n* = 11)	92% (*n* = 12)
9. A service is required to manage bowel problems	67% (*n* = 12)	91% (*n* = 11)	92% (*n* = 12)
10. A service is required to manage pain caused by bone metastases	78% (*n* = 11)	70% (*n* = 10)	100% (*n* = 11)
11. Patient diagnosis should be given by a nurse	56% (*n* = 16)	56% (*n* = 9)	83% (*n* = 12)
12. Treatment options should be discussed with a patient in the first instance by a nurse	44% (*n* = 16)	50% (*n* = 10)	82% (*n* = 11)
13. The diagnostic pathway should be nurse-led	73% (*n* = 15)	100% (*n* = 12)	80% (*n* = 10)
14. Pre-biopsy counselling should be nurse-led	93% (*n* = 14)	-	91% (*n* = 11)
15. With the exception of the initial post-operative review, post-operative care should be nurse-led	-	75% (*n* = 8)	83% (*n* = 6)
16. Radiotherapy follow-up should be nurse-led	87% (*n* = 15)	-	83% (*n* = 12)
17. Surgical follow-up should be nurse-led	64% (*n* = 14)	75% (*n* = 8)	90% (*n* = 10)
18. Side effects services should be nurse led	69% (*n* = 11)	100% (*n* = 10)	92% (*n* = 12)
19. There is a need to develop a nurse specialist role to support patients with symptoms, side effects and treatment queries on an as and when required basis	81% (*n* = 16)	**-**	100% (*n* = 12)
20. Side effects services should be nurse led	69% (*n* = 11)	100% (*n* = 10)	92% (*n* = 12)
21. Training programmes for nurse specialists should be developed	80% (*n* = 15)	-	100% (*n* = 12)
22. Nurses should be trained through networking with individuals in similar posts	93% (*n* = 15)	-	100% (*n* = 12)
23. Prostate cancer services require the development of a Scotland-wide nursing and allied health professional working group	-	-	100% (*n* = 12)

**Table 3:** Consensus on other capacities (primary care) to support service development.

Statement	Round 1 (*n* = 16)	Round 2 (*n* = 13)	Round 3 (*n* = 12)
Statement accepted			
1. Primary care follow-up would create a need for clinic correspondence management	85% (*n* = 13)	-	-
2. A service should be provided in primary care for…			
a….supporting weight management	100% (*n* = 15)	-	80% (*n* = 10)
b….supporting diabetes	100% (*n* = 15)	-	80% (*n* = 10)
c….cholesterol monitoring	100% (*n* = 14)	-	80% (*n* = 10)
d. …blood pressure monitoring	100% (*n* = 14)	-	80% (*n* = 10)
3. A service should be provided in the community for…			
a….long term hormone therapy prescription	88% (*n* = 16)	-	100% (*n* = 12)
b. …neo-adjuvant hormone injection administration	100% (*n* = 16)	-	-
c. …long term hormone injection administration	100% (*n* = 16)	-	-
d. …patient support when receiving hormone injections	67% (*n* = 12)	82% (*n* = 11)	-
e….oral drug dispension	100% (*n* = 13)	-	100% (*n* = 12)
4. Primary care practitioners should be included within [national] prostate cancer working groups for the develop, 9pment and implementation of community-based interventions and services	-	-	100% (*n* = 12)
Dissensus			
5. A service should be provided in the community for a uro-oncology nurse injection service to provide long term and neo-adjuvant hormone therapy as well as support to these patients	-	-	45% (*n* = 11)
6. Prostate cancer specialists and primary care should work side by side when following men up	79% (*n* = 14)	-	-
7. The follow-up of post-prostatectomy should take place in primary care with a return visit to secondary care following treatment	-	40% (*n* = 10)	56% (*n* = 9)
8. The follow-up of post-radiotherapy should take place in primary care with a return visit to secondary care	27% (*n* = 15)	-	-
9. The follow-up of post-radiotherapy should take place in primary care with a return visit to secondary care following treatment	-	50% (*n* = 12)	33% (*n* = 12)
10. Long term hormone therapy should be…			
a. … nurse-led	100% (*n* = 15)	62% (*n* = 13)	50% (*n* = 12)
b. … GP-led	-	39% (*n* = 13)	50% (*n* = 12)
11. The follow-up of patients’ receiving long term hormone therapy should take place in primary care only with the presence of rapid access mechanisms feeding back into secondary care	50% (*n* = 16)	-	-
Statement rejected			
12. GPs are equipped to counsel men effectively on PSA testing at diagnosis	57% (*n* = 14)	33% (*n* = 9)	18% (*n* = 11)
13. The follow-up of active surveillance should take place in primary care…			
a. … by GPs	0% (*n* = 15)	-	-
b. … by practice nurses	0% (*n* = 15)	-	-
c. … by specialists	7% (*n* = 15)	-	-
d. … only with the presence of rapid access mechanisms feeding back into secondary care	13% (*n* = 15)	-	-
e. … with a return visit to secondary care	40% (*n* = 15)	23% (*n* = 13)	8% (*n* = 12)
14. Secondary care holds onto patients too long after treatment	25% (*n* = 12)	17% (*n* = 12)	-
15. The follow-up of post-prostatectomy should take place in primary care…			
a. … by GPs	7% (*n* = 14)	-	-
b. … by practice nurses	0% (*n* = 14)	-	-
c. … by specialists	7% (*n* = 14)	-	-
d. … only with the presence of rapid access mechanisms feeding back into secondary care	36% (*n* = 14)	-	-
… … following treatment	-	0% (*n* = 10)	-
… … prior to discharge	-	11% (*n* = 9)	-
e. … with a return visit to secondary care	20% (*n* = 14)	-	-
… … prior to discharge	-	11% (*n* = 9)	-
16. The follow-up of post-radiotherapy should take place in primary care…			
a. … by GPs	0% (*n* = 15)	-	-
b. … by practice nurses	0% (*n* = 15)	-	-
c. … by specialists	0% (*n* = 15)	-	-
d. … only with the presence of rapid access mechanisms feeding back into secondary care	33% (*n* = 15)	-	-
… … following treatment	-	0% (*n* = 12)	-
… … prior to discharge	-	27% (*n* = 11)	0% (*n* = 12)
e. The follow-up of post-radiotherapy should take place in primary care with a return visit to secondary care prior to discharge	-	0% (*n* = 11)	-
17. The follow-up of patients’ receiving long term hormone therapy should take place in primary care…			
a. … by GPs	13% (*n* = 16)	-	-
b. … by practice nurses	13% (*n* = 16)	-	-
c. … by [prostate cancer] specialists	13% (*n* = 16)	-	-
d. … with a return visit to secondary care	13% (*n* = 16)	-	-

**Table 4:** Consensus on other capacities (patient choice and responsibilities) to support service development.

Statement	Round 1 (*n* = 16)	Round 2 (*n* = 13)	Round 3 (*n* = 12)
Statements accepted			
1. Pre-biopsy counselling is required to discuss pros and cons of biopsy	92% (*n* = 13)	-	91% (*n* = 11)
2. Initial treatment discussions are required to discuss treatment plans with patients receiving palliative care	92% (*n* = 12)	-	100% (*n* = 12)
3. Orchidectomy should be discussed as a treatment option with patients	90% (*n* = 10)	-	100% (*n* = 12)
4. Patients should be given more responsibility during follow-up	83% (*n* = 12)	-	-
5. The follow-up of post-radiotherapy patients after functional outcomes are achieved, should led by secondary care and utilise virtual clinics	-	36% (*n* = 11)	100% (*n* = 12)
Dissensus			
6. The follow-up of post-radiotherapy patients…			
a …should table place in secondary care only	60% (*n *= 15)	-	-
b …prior to functional outcomes being achieved, should take place under current mechanisms of follow-up	-	25% (*n* = 12)	58% (*n* = 12)
7. The follow-up of post-prostatectomy patients…			
a. …should take place in secondary care only	50% (*n* = 14)	-	-
b. …after functional outcomes are achieved, should be led by secondary care and utilise virtual clinics	-	33% (*n* = 9)	78% (*n* = 9)
c. …prior to functional outcomes being achieved, should be led by secondary care and utilise virtual clinics	-	30% (*n* = 10)	33% (*n* = 9)
Statements rejected			
8. The follow-up of active surveillance should involve patients self-monitoring their PSA level	27% (*n* = 15)	-	-
a. Active surveillance follow-up should be led by secondary care via patients self-monitoring PSA with contact details of secondary care staff for concerns	-	0% (*n* = 13)	-
b. Active surveillance follow-up should be led by secondary care via patients self-monitoring PSA and a return visit to secondary care	-	15% (*n* = 13)	-
9. The follow-up of post-prostatectomy patients should involve patients self-monitoring their PSA level	43% (*n* = 14)	-	-
a. During the initial period following radical prostatectomy, follow-up should be led by secondary care with patient self-monitoring PSA	-	20% (*n* = 10)	0% (*n* = 9)
…if patients should self-monitor their PSA, this should be patient led with contact details of secondary care staff if concerns arise	-	56% (*n* = 9)	-
…if patients should self-monitor their PSA, this should be with return visits to secondary care	-	33% (*n* = 9)	-
…if patients should self-monitor their PSA, this should be with regular telephone review	-	11% (*n* = 9)	-
b. Prior to discharge, follow-up for patients who have undergone radical prostatectomy should be led by secondary care with patient self-monitoring PSA	-	71% (*n* = 9)	11% (*n* = 9)
…if patients should self-monitor their PSA, this should be patient led with contact details of secondary care staff if concerns arise	-	14% (*n* = 7)	-
…if patients should self-monitor their PSA, this should be with return visits to secondary care	-	14% (*n* = 7)	-
…if patients should self-monitor their PSA, this should be with regular telephone review	-	11% (*n* = 9)	-
10. The follow-up of post-radiotherapy patients should involve patients self-monitoring their PSA level	27% (*n* = 15)	-	-
a. During the initial period following radiotherapy, follow-up should be led by secondary care with patient self-monitoring PSA	-	9% (*n* = 12)	-
…if patients should self-monitor their PSA, this should be patient led with contact details of secondary care staff if concerns arise	-	56% (*n* = 9)	-
…if patients should self-monitor their PSA, this should be with return visits to secondary care	-	22% (*n* = 9)	-
…if patients should self-monitor their PSA, this should be with regular telephone review	-	22% (*n* = 9)	-
b. Prior to discharge, follow-up for patients who have undergone radiotherapy should be led by secondary care with patient self-monitoring PSA	-	27% (*n* = 11)	0% (*n* = 12)
…if patients should self-monitor their PSA, this should be patient led with contact details of secondary care staff if concerns arise	-	50% (*n* = 10)	-
…if patients should self-monitor their PSA, this should be with return visits to secondary care	-	30% (*n* = 10)	-
…if patients should self-monitor their PSA, this should be with regular telephone review	-	20% (*n* = 10)	-
11. The follow-up of patients taking neo-adjuvant hormone therapy should involve patients self-monitoring their PSA level	13% (*n* = 15)	-	-
12. The follow-up of post-radiotherapy patients…			
a …prior to functional outcomes being achieved, should be led by secondary care and utilise virtual clinics	-	17% (*n* = 12)	-
b …after functional outcomes are achieved, should take place under current mechanisms of follow-up	-	9% (*n* = 11)	**-**
c …after functional outcomes are achieved, should be led by secondary care with telephone review	-	0% (*n* = 11)	-
13. The follow-up of post-prostatectomy patients…			
a. …prior to functional outcomes being achieved, should take place under current mechanisms of follow-up	-	10% (*n* = 10)	-
b. … after functional outcomes are achieved, should take place under current mechanisms of follow-up	-	22% (*n* = 9)	11% (*n* = 9)
c. …after functional outcomes are achieved, should be led by secondary care with telephone review	-	0% (*n* = 10)	-

#### 3.3.1. Leadership and cohesion

Healthcare professionals rejected the current model of Health Board-led care (Table 3, Statement 12) in place of a centralised leadership structure (Table 1, Statement 9–10) and consensus was reached for the further development of MCNs (Table 1, Statements 1–2). However, healthcare professionals were divided over the need for specialisms to work together (Table 1, Statement 11) though consensus was reached for improved methods of integration and communication between specialities (Table 1, Statement 3). In R3, healthcare professionals were asked to consider mechanisms for service development and consensus was reached for all of these: national, speciality-specific working groups are needed to design quality care and communicate this with MCNs, with MCNs responsible for the regional, multidisciplinary implementation of quality care (Table 1, Statements 4–8).

#### 3.3.2. Recruitment and training

Healthcare professionals reached consensus for the further development of specialist nurse roles across the Service, including the development of multiple nurse-led services (Table 2, Statements 1–19). Training of specialist nurses and allied health professionals should be multi-faceted; nurses should be trained through engaging with a training programme, networking with individuals in similar posts, and supported through the development of a national nursing and allied health professional working group (Table 2, Statements 20–22).

#### 3.3.3. Other capacities

Healthcare professionals reached consensus that primary care practitioners did not have the capabilities to counsel men in relation to most aspects of prostate cancer care including PSA testing at diagnosis and treatment follow-up (Table 3, Statements 6–17), and moving services to primary care would create increased workload in secondary care through correspondence management (Table 3, Statement 1). Support for developing the role of primary care was most evident in the delivery of palliative care pathways and for tertiary health promotion purposes (Table 3, Statements 2–3, 5), though healthcare professionals were divided over who should then be ultimately responsible for the management of this patient population (Table 3, Statements 10). In R3, healthcare professionals reached consensus that primary care practitioners should be included within national prostate cancer working groups providing opportunities to explore capacity development needs jointly going forward (Table 3, Statement 4).

Further, whilst healthcare professionals were keen to improve opportunity for patients to have increased autonomy and responsibility in their own care (Table 4, Statements 1–4), healthcare professionals rejected that patients should have a role in monitoring their own Prostate Specific Antigen (PSA) levels even with regular specialist support (Table 4, Statements 5–13). Rather, consensus was reached for follow-up to remain in secondary care (Table 4, Statements 5–13) possibly with use of virtual clinics (Table 4, Statement 5).

## 4. Discussion

### 4.1. Wider context

This study drew on expertise from within a national cancer service that had been unable to develop to meet demand. Interviews found that reform was needed within prostate cancer services in Scotland as services had been “*redesigned as much as [they] probably [could], and we’re struggling”* (Participant 1), and identified tensions between healthcare professionals and policy makers rooted in different understandings of quality care. Further, a lack of leadership of service developments nationally and regionally; cohesive working; recruitment and training of nurse specialists; and other capacities to meet demand, were identified as barriers to service development. Healthcare professionals identified a need for national, multidisciplinary working groups to develop capacity and meet demand.

Results from this study shed light on important barriers and facilitators in health service development. Whilst it is common for tensions to exist between policy-makers and those delivering health services (30–33), this study adds to the growing body of evidence that top-down approaches to the development of strategic visions for healthcare hinder service development. Collaboration between policy makers and healthcare professionals promise visions ready for application to health service development contexts (34). Braithwaite (35) describes change in health services as moving “*to its own rhythm*” (pp. 1) and advocates that services need to be empowered to realise change.

The use of working groups or networks have been an important mechanism in facilitating service development throughout the COVID-19 pandemic (4, 36, 37). As such, findings are consistent with other studies which found that leadership of service development nationally do not require traditional healthcare leadership styles and structures (38, 39). Rather, leadership of national service developments should be systemic, service-wide processes based on shared values (40, 41), where a group works to coordinate development towards a new vision (41). Working groups have been found to lead to greater degree of structural and psychological empowerment within the services through encouraging innovation in development (42–45). Importantly, findings recognised the need for different “levels” of working groups with clear terms of reference: higher level groups to set the standard for quality care, and regional groups to implement this standard within the local context.

Further, whilst there are many ways of fostering collaboration to support service development, such as through team building, organisational strengthening and relationship building, cohesion was the model described by participants. Cohesion refers to the strength of relationships and solidarity within a community (46, 47) or the unitedness of a group in obtaining its goal or purpose (48, 49) and includes factors such as interpersonal trust, norms of reciprocity and mutual aid (46, 50–53). Studies have found that high cohesion relates to the absence of latent conflict from longstanding difference that can result from inequities (46) or functional diversity (49), and functional diversity has been found to have a curvilinear relationship with cohesion, where little diversity and high diversity can strengthen cohesion (50–55). In health service development, cohesion has been found to support investment in group activities (53–55) and working towards a shared vision (54, 55).

Consensus reached reflects wider literature (56–65) that identifies support for shift of prostate cancer care services to primary care only where healthcare professionals working in primary care are perceived to have the capacities to best meet patient need when compared with secondary care, though wider literature relating to care for patients who are palliative relates to end of life care only (59–65). With healthcare professionals struggling to realise the widespread shift given current capacities in primary care, and the imperative to deliver specialist care locally has had a substantial impact on the wellbeing of healthcare professionals working in primary care and has led to widespread recruitment concerns (66, 67). Whilst shifting care to primary care is a key policy objective of Scottish health and social care policy (11, 13, 68–70), findings highlight a need for reconsideration of widespread integration of healthcare services considering the new health and social care landscape and echo the Christie commission's (71) emphasis on the development of function-led over form-led care as well as Weir's (72) caution that shifting care to primary care was not a solution for all service delivery issues.

Whilst there is a plethora of literature advocating for participation of patients in their own care including the Patient Rights (Scotland) Act (73), Scottish health policy, the Christie Commission report (72), and evidence showing improvements in measures of care quality including survival (74–76), healthcare professionals were hesitant to use patient capacities to meet demand. The right of a patient to participate as fully as possible in their own care has been identified as a pertinent area of reform in Scottish healthcare in the last 10 years (77, 78). However, practical guidance supporting healthcare professionals to ethically translate this into practice is limited. Recent recommendations for change in Scottish cancer policy identified a need for clearer guidance on use of patient capabilities within cancer services to inform service development (79).

### 4.2. Strengths and limitations

Healthcare professional expertise is an asset in health service development (80, 81). This study exploited existing expertise of service need across a multi-disciplinary sample of healthcare professionals working across Scotland to reach consensus on how a national cancer service should be developed to meet demand. Apart from two GPs who participated in interviews, all healthcare professionals were prostate cancer specialists and so expertise was limited by lack of exposure to good practice in service development in other specialities. While it is not possible to know the total potential sample size for this study, this study represents a small, but diverse population of relevant healthcare professionals.

### 4.3. Implications

Findings from this study highlight a need for policy makers to work cohesively with healthcare professionals. Wider literature shows that where healthcare professionals are not supported by policy makers, potential for service development is likely to be limited (80–83). Findings also provide policy makers and healthcare professionals with consensus on the need for national, multidisciplinary working groups, which will facilitate capacity development across services. Further, the development of working groups provide opportunity for coproduction of services with varied stakeholder involvement (82, 83), and it has been found cohesive working within groups supports development and implementation of shared visions for health services (54, 55) and so have potential to support services to overcome further barriers to development.

## 5. Conclusions

As health systems recover and remobilise towards a “new normal” in health service delivery, understanding of barriers and facilitators to service development within already under pressure services is important. This work highlights how lack of shared vision between policy makers and healthcare professionals can hinder service development and identifies key mechanisms to support service change.

## Data Availability

The raw data supporting the conclusions of this article will be made available by the authors, without undue reservation.
